# Multi-Level Deep Deterministic Policy Gradient reinforcement learning for safety-constrained trajectory optimization of collaborative industrial robots

**DOI:** 10.3389/frai.2026.1914042

**Published:** 2026-10-09

**Authors:** Akhanseri Ikramov, Sayat Ibrayev

**Affiliations:** 1 Laboratory of Mechanics of Robots and Manipulators, Joldasbekov Institute of Mechanics and Engineering, Almaty, Kazakhstan; 2 Al-Farabi Kazakh National University, Almaty, Kazakhstan

**Keywords:** autonomous navigation, collaborative robots, DDPG, industrial robotics, motion planning, reinforcement learning, trajectory planning

## Abstract

Collaborative robots operating in modern manufacturing environments require trajectory planning strategies that simultaneously support efficiency, adaptability, and operational safety. This study proposes a Multi-Level Deep Deterministic Policy Gradient (ML-DDPG) framework for safety-constrained trajectory optimization. The framework combines a strategic waypoint level, a continuous DDPG motion-control policy, and a reactive feasibility projection that corrects unsafe actions before execution. The trajectory-planning problem is formulated as a Markov Decision Process with reward terms for goal progress, obstacle clearance, smoothness, energy, execution time, and safety. Descriptive experiments in a custom PyTorch-based three-dimensional environment compare ML-DDPG with conventional planners and the DDPG, TD3, and SAC baselines. In the retained single training run, ML-DDPG produced observed values of 98.7% success, 1.08 mm trajectory error, a 0.98 smoothness index, and no recorded collision. These values are single-run observations and are not means over independent seeds; variability and statistical significance cannot be estimated from the available record. The exported traces show faster reward growth within the 200-episode budget, and the single-run ablation record associates the largest observed gains with the combined multi-level, reward, and projection components. One UR5e demonstration yielded 0.018 m mean tracking error, 0.042 m maximum tracking error, and no collision in that trial. A separate ABB welding sequence is included only as qualitative feasibility evidence because synchronized trajectory, force, and weld-quality logs were unavailable. The findings motivate further evaluation, while multi-seed training, repeated hardware trials, complete reporting of evaluation procedures, and cross-platform validation remain necessary before broader generalization.

## 1 Introduction

Collaborative robots have become a key technology in modern intelligent manufacturing systems due to their ability to operate safely with human workers while providing high flexibility, precision, and automation efficiency. Unlike conventional industrial robots that execute predefined repetitive tasks in isolated environments, collaborative robots require adaptive motion planning capabilities to respond to dynamic workspaces, changing production requirements, and unexpected obstacles. Kim et al. (2024) highlighted that the integration of artificial intelligence into collaborative robotics has significantly improved autonomy, perception, and decision-making capabilities in industrial applications. However, generating optimal trajectories that simultaneously satisfy accuracy, safety, energy efficiency, and real-time constraints remains a major challenge for advanced robotic systems.

Traditional trajectory planning includes graph search, sampling-based planning, interpolation, and optimization-based control. The A* algorithm was introduced by Hart et al. (1968), while asymptotically optimal sampling-based planning was formalized for RRT* by Karaman and Frazzoli (2011). These approaches remain reliable in structured workspaces, but their performance depends on environmental models and online replanning capacity. Recent spline and model-predictive methods can produce smooth industrial manipulator trajectories, although additional mechanisms are still required for dynamic constraints and changing obstacle configurations (Momynkulov et al., 2025).

Deep reinforcement learning provides an alternative for continuous robotic control. Deep Deterministic Policy Gradient (DDPG) was introduced by Lillicrap et al. (2016) as an off-policy actor-critic method that combines deterministic policy gradients, replay memory, and target networks. TD3 later reduced function-approximation error through twin critics and delayed policy updates (Fujimoto et al., 2018), while SAC introduced entropy-regularized stochastic control (Haarnoja et al., 2018). These methods are relevant to collaborative robots because joint and Cartesian commands are continuous.

Practical deployment nevertheless remains constrained by unsafe exploration, sensitivity to initialization, imperfect simulator dynamics, and limited evidence across hardware platforms. Constrained policy optimization and action-layer correction offer complementary ways to restrict unsafe behavior (Achiam et al., 2017; Dalal et al., 2018). Recent industrial studies have also emphasized adaptive safety in human-robot workspaces (Feng et al., 2025), while a current survey of real-world robotic reinforcement learning identifies reproducibility and sim-to-real robustness as persistent challenges (Tang et al., 2025). Reinforcement-learning-based admittance control has also been investigated for physical human-robot interaction under output constraints (Gao et al., 2025).

To address these limitations, this study proposes a Multi-Level Deep Deterministic Policy Gradient reinforcement learning framework for safety-constrained trajectory optimization of collaborative industrial robots. The proposed approach integrates hierarchical decision-making, safety-aware reward optimization, adaptive exploration mechanisms, and simulation-to-real transfer strategies. The framework is designed to generate smooth, efficient, and collision-free trajectories while maintaining real-time feasibility for industrial operations. Experimental validation demonstrates the effectiveness of the proposed method in achieving autonomous trajectory planning and improving the adaptability of collaborative robots for intelligent manufacturing applications.

## 2 Related works

Classical motion planning provides transparent feasibility guarantees when the environment is sufficiently known. A* performs heuristic graph search (Hart et al., 1968), whereas RRT* converges asymptotically toward an optimal path under its sampling assumptions (Karaman and Frazzoli, 2011). Interpolation and spline methods can improve geometric continuity, but do not by themselves learn how to trade task progress against changing safety risk.

Recent industrial trajectory studies combine learning with optimization to improve responsiveness. Simulation-efficient deep reinforcement learning has been evaluated for manipulator trajectory planning (Zhao et al., 2025), and hierarchical reinforcement learning with explicit path optimization has been studied for adaptive robotic-arm obstacle avoidance (Ma et al., 2026). These studies motivate structured decision making, but they do not eliminate the need to define the hierarchy, information flow, and safety mechanism precisely.

Reinforcement learning is formalized as sequential return maximization under a Markov Decision Process (Sutton and Barto, 2018). Hierarchical reinforcement learning extends this formulation by introducing temporally extended actions or decomposed decision layers. The options framework defines initiation sets, internal policies, and termination conditions (Sutton et al., 1999), while broader hierarchical methods separate decisions across temporal or task abstractions (Barto and Mahadevan, 2003).

Deep Q-Networks demonstrated value-function approximation for discrete action spaces (Mnih et al., 2015), but manipulator control normally requires continuous actions. DDPG addresses this requirement through a deterministic actor and learned critic (Lillicrap et al., 2016). In the present framework, DDPG is the only learned control policy; the strategic and reactive levels are deterministic modules surrounding that policy rather than three separately trained agents.

The continuous-control baselines used in this study are attributed to their original sources. TD3 uses clipped double-Q targets, delayed actor updates, and target-policy smoothing (Fujimoto et al., 2018). SAC maximizes expected return and policy entropy (Haarnoja et al., 2018). PPO applies a clipped surrogate objective for stable on-policy updates (Schulman et al., 2017), but it is discussed only as related work because PPO was not included in the reported experiment table.

Phase-based and hierarchical robotic learning has previously decomposed long-horizon tasks into ordered stages. For example, reinforcement-learning motion planning for robotic bricklaying divides a collaborative-arm task into task-specific phases (Navazani et al., 2026). The present ML-DDPG differs in that its strategic level continuously supplies intermediate waypoints, the tactical level uses one shared continuous policy, and the reactive level projects every raw action into a feasible set; it does not train an independent policy for each phase. Reinforcement learning has also been applied to human-robot collaborative assembly (Zhang et al., 2022), and large language models have been used to generate reward functions for robotic manipulation (Turcato et al., 2025).

Safe reinforcement learning can enforce constraints through the objective, the policy update, or a separate action-correction layer. Constrained policy optimization targets expected-cost bounds (Achiam et al., 2017), whereas a safety layer can minimally modify an action before execution (Dalal et al., 2018). Recent human-robot research has combined adaptive perception and deep reinforcement learning for dynamic industrial safety (Feng et al., 2025), reinforcing the need to report both learned and non-learned safeguards.

Accordingly, the contribution claimed here is not hierarchical decomposition alone. It is the integration of continuous waypoint conditioning, a shared DDPG actor-critic controller, six-term task-and-safety reward shaping, and a per-step constrained projection within one trajectory-planning pipeline, followed by simulation, UR5e tracking, and a separate qualitative welding demonstration. This positioning narrows the novelty claim and distinguishes the architecture from option-based, multi-policy, and fixed phase-based formulations.

## 3 Materials and methods

The ML-DDPG framework was implemented in PyTorch within a custom three-dimensional kinematic and dynamic simulation environment configured around a six-degree-of-freedom UR5e collaborative manipulator. The method combines a deterministic strategic waypoint generator, one continuous DDPG actor-critic controller, and a non-learning safety projection. This definition clarifies which components are trained and how the hierarchy is executed.

### 3.1 Overview of the proposed Multi-Level DDPG framework

This study proposes a Multi-Level Deep Deterministic Policy Gradient (ML-DDPG) framework for safety-constrained trajectory optimization of collaborative industrial robots. The main objective of the proposed approach is to generate adaptive, smooth, and collision-free trajectories by integrating hierarchical decision-making, continuous control optimization, and safety-aware learning. The trajectory optimization problem is formulated as a reinforcement learning process, where the collaborative robot interacts with the industrial environment and progressively improves its control strategy based on accumulated experience.

Figure 1 represents the overall workflow of the proposed ML-DDPG framework, including industrial environment modeling, Markov Decision Process (MDP) formulation, hierarchical decision-making, DDPG-based policy optimization, trajectory generation, simulation validation, and real-world deployment. The framework starts by collecting environmental information, including robot states, target positions, obstacle locations, and workspace constraints. Based on this information, the reinforcement learning agent generates optimized actions while satisfying predefined safety and operational limitations.

**Figure 1:**
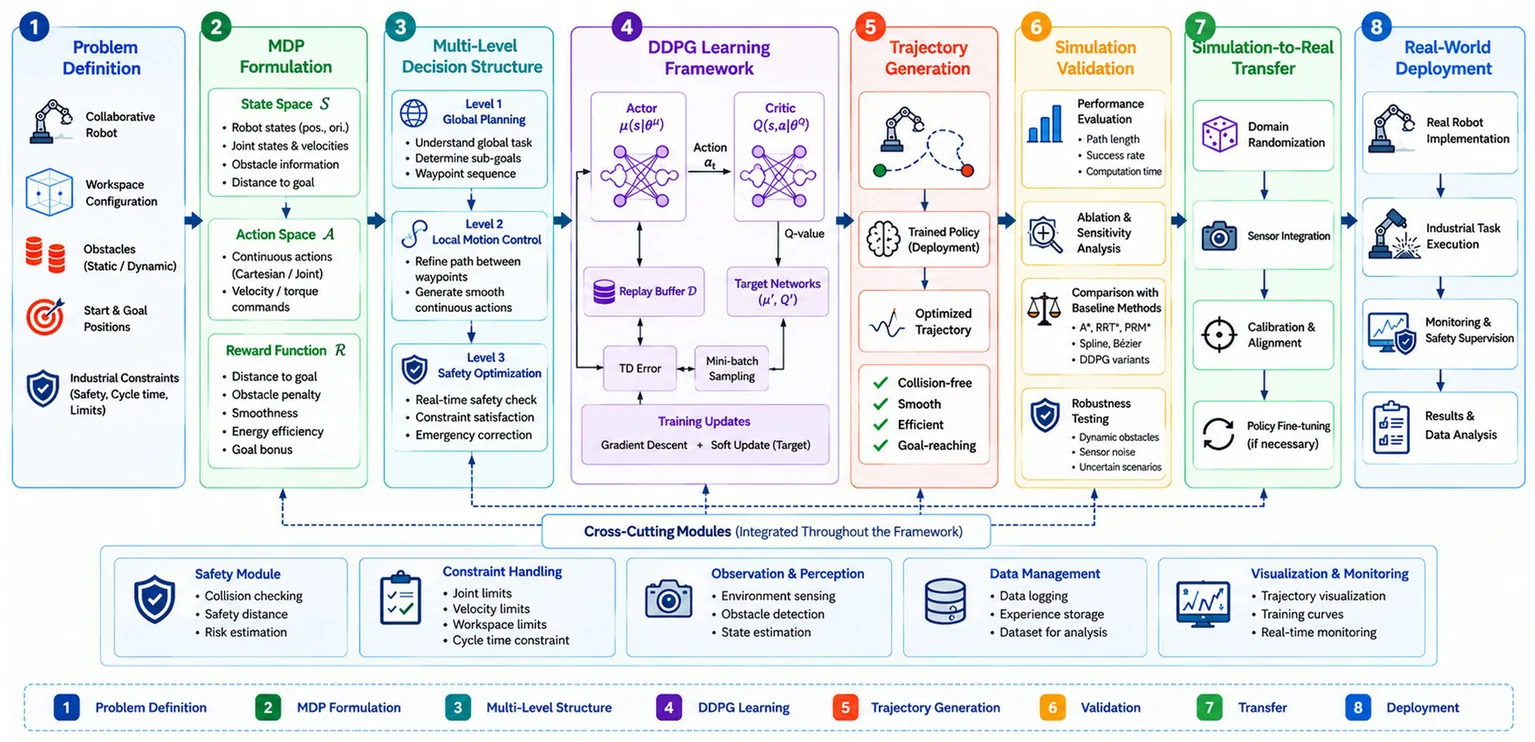
Flowchart of the proposed multi-level DDPG framework for safety-constrained trajectory optimization of collaborative robots.

The trajectory planning problem is defined as an MDP represented by:


M=(S,A,P,R,γ)

where S denotes the state space, A represents the continuous action space, P defines the transition probability between states, R is the reward function, and γ represents the discount factor. At each time step t , the agent observes the current state $s_{t}$ , performs an action $a_{t}$ , receives reward $r_{t}$ , and moves to the next state $s_{t+1}$ .

### 3.2 State and action space definition

The state representation is designed to include essential information required for adaptive robotic motion planning. The state vector consists of robot kinematic information, environmental conditions, target information, and previous control actions. The complete state representation is formulated as:


$$s_{t}=[p_{t},q,{\dot{q}}_{t},o_{t},d_{t},a_{t-1}]$$

where $p_{t}$ represents the end-effector position, $q_{t}$ indicates joint positions, ${\dot{q}}_{t}$ denotes joint velocities, $o_{t}$ represents obstacle information, $d_{t}$ defines the distance between the robot and target, and $a_{t-1}$ represents the previous action. This formulation enables the agent to understand both current robot conditions and environmental variations during trajectory execution.

The action space is defined as a continuous control vector that determines robot movement commands. The generated action is formulated as:


$$a_{t}=[\Delta x_{t},\Delta y_{t},\Delta z_{t},\Delta q_{t}]$$

where $\Delta x_{t}$ , $\Delta y_{t}$ , and $\Delta z_{t}$ represent Cartesian displacement commands, while $\Delta q_{t}$ corresponds to joint adjustment values. The continuous action representation enables smooth trajectory generation compared with discrete action-based reinforcement learning methods.

### 3.3 Multi-Level DDPG agent architecture

The proposed architecture contains three execution levels. Level 1 is a strategic waypoint generator that converts the goal and obstacle map into the next intermediate Cartesian target. Level 2 is the learned tactical controller: a single DDPG actor maps the augmented state and current waypoint to a raw continuous action. Level 3 is a reactive, non-learning safety layer that tests joint, velocity, workspace, and obstacle-clearance constraints and minimally corrects an infeasible action before it reaches the robot controller. Figure 2 depicts these roles and the shared replay-based update.

**Figure 2:**
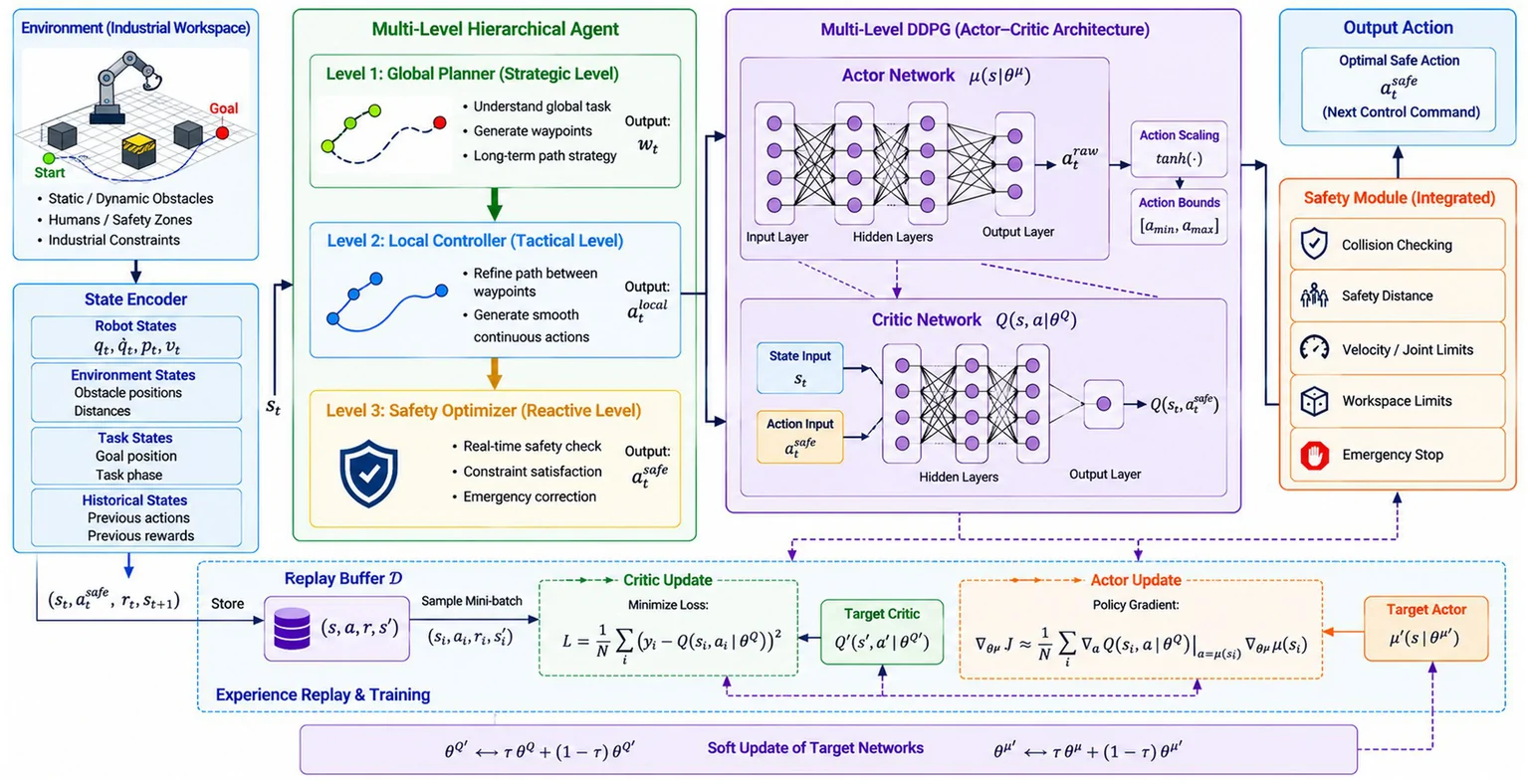
Architecture of the proposed Multi-Level DDPG agent.

Information flows sequentially as $s_{t}\to g_{t}\to [s_{t},g_{t}]\to a_{t}\to a_{t}^{\text{safe}}\to ℰ$ . Where, $s_{t}$ —current state, $g_{t}$ —current waypoint (goal), $\left[s_{t},g_{t}\right]$ —augmented state, $a_{t}$ —raw action generated by the policy, $a_{t}^{\text{safe}}$ —safety-projected action, ℰ —environment.

The transition stored in replay memory contains the executed projected action, not the rejected raw proposal. Only the actor, critic, and their target networks are optimized; the waypoint and projection modules are deterministic. Consequently, the term multi-level describes a coupled execution hierarchy rather than three independently trained policies.

Both actor and critic use three fully connected hidden layers of 256, 256, and 128 units with ReLU activations. The actor terminates in a tanh output scaled to the admissible command range, and the critic terminates in a linear scalar *Q* value. The architecture was selected as a capacity-latency compromise for the reported state vector and was held fixed across DDPG-family comparisons. No automated hyperparameter search was performed; the learning rates, replay size, batch size, discount factor, and soft-update coefficient in Table 1 were fixed before the reported comparisons.

**Table 1:** Experimental configuration and hyperparameters of the proposed ML-DDPG framework.

Category	Parameter	Value
Environment	Workspace	3D industrial environment
	Obstacles	Static and dynamic
	Task	Trajectory optimization
State space	Inputs	Position, velocity, obstacle distance, goal distance
Action space	Action type	Continuous Cartesian control
Network architecture	Actor/critic layers	256–256–128
	Activation function	ReLU
Training parameters	Episodes	200
	Max steps/episode	500
	Batch size	64
	Replay buffer	1 × 10^6^
	Actor learning rate	1 × 10^−4^
	Critic learning rate	1 × 10^−3^
	Discount factor (gamma)	0.99
	Soft update (tau)	0.001
Exploration	Noise model	Ornstein–Uhlenbeck
Safety constraints	Collision avoidance	Enabled
	Joint limits	Enabled
	Velocity limits	Enabled
	Workspace limits	Enabled
Evaluation metrics	Metrics	Success Rate, Reward, Error, Collision Rate, Smoothness
Hardware	GPU	NVIDIA RTX 4070
	Memory	32 GB RAM
	Framework	PyTorch
Environment	Simulation platform	Custom PyTorch-based 3D environment
Robot platforms	Navigation/tracking	UR5e collaborative robot
	Welding demonstration	Separate ABB six-axis manipulator; exact model to be confirmed
Reward design	Goal reward weight ( $w_{\text{goal}}$ )	**1.0**
	Obstacle reward weight ( $w_{\mathrm{obs}}$ )	**0.8**
	Smoothness reward weight ( $w_{\text{smooth}}$ )	**0.5**
	Energy reward weight ( $w_{\text{energy}}$ )	**0.2**
	Time reward weight ( $w_{\text{time}}$ )	**0.2**
	Safety reward weight ( $w_{\text{safe}}$ )	**1.2**
	Penalty coefficient ( λ )	**10**

Bold values indicate the six fixed reward weights and the safety penalty coefficient used in the reported experiments.

For state dimension $d_{s}$ and action dimension $d_{a}$ , one actor inference has complexity $O(d_{s}\cdot 256+256^{2}+256\cdot 128+128\cdot d_{a})$ ; $d_{s}$ is the state dimension, $d_{a}$ is the action dimension, $256^{2}$ corresponds to the hidden-to-hidden fully connected layer, 256·128 corresponds to the second hidden layer, $128\cdot d_{a}$ corresponds to the output layer mapping to the action space. The critic adds the corresponding state-action input and a scalar output. Replay sampling is O(B), and each gradient update is linear in batch size B and network parameter count. The measured planning latency reported in Table 2 is 17.1 ms for ML-DDPG on the stated RTX 4070 platform; this is a descriptive device-specific measurement rather than a hardware-independent real-time guarantee.

**Table 2:** Single-run quantitative comparison of trajectory optimization methods.

Method	Path length (m)	Trajectory error (mm)	Smoothness index ↑	Execution time (ms)	Collision rate (%)	Success rate (%)
Linear interpolation	1.62	8.47	0.71	4.2	18.3	72.4
Bézier curve	1.58	6.95	0.82	5.8	12.6	80.7
B-spline	1.55	5.84	0.89	6.7	8.9	86.2
A* search	1.49	4.96	0.76	15.4	6.8	89.4
RRT*	1.46	4.18	0.81	24.8	5.2	91.8
DDPG	1.38	3.42	0.91	18.7	3.6	93.1
TD3	1.34	2.67	0.94	19.3	2.1	95.4
SAC	1.36	2.91	0.93	20.5	2.8	94.6
Proposed ML-DDPG	1.27	1.08	0.98	17.1	0.0	98.7

The DDPG agent consists of an actor network and a critic network. The actor network generates deterministic control actions based on the current state:


$$a_{t}=\mu (s_{t}|\theta^{\mu })$$

where μ represents the actor policy and $\theta^{\mu }$ indicates actor network parameters. The critic network estimates the expected return of the selected action using:


$$Q=Q(s_{t},a_{t}|\theta^{Q})$$

The learning procedure is summarized in Algorithm 1. At each step, the strategic level updates the local waypoint, the actor proposes a continuous command, the safety layer projects it to the feasible set, and the executed transition is used for critic and actor updates following DDPG (Lillicrap et al., 2016).

Algorithm 1 **Proposed Multi-Level DDPG Algorithm for safety-constrained trajectory optimization.**

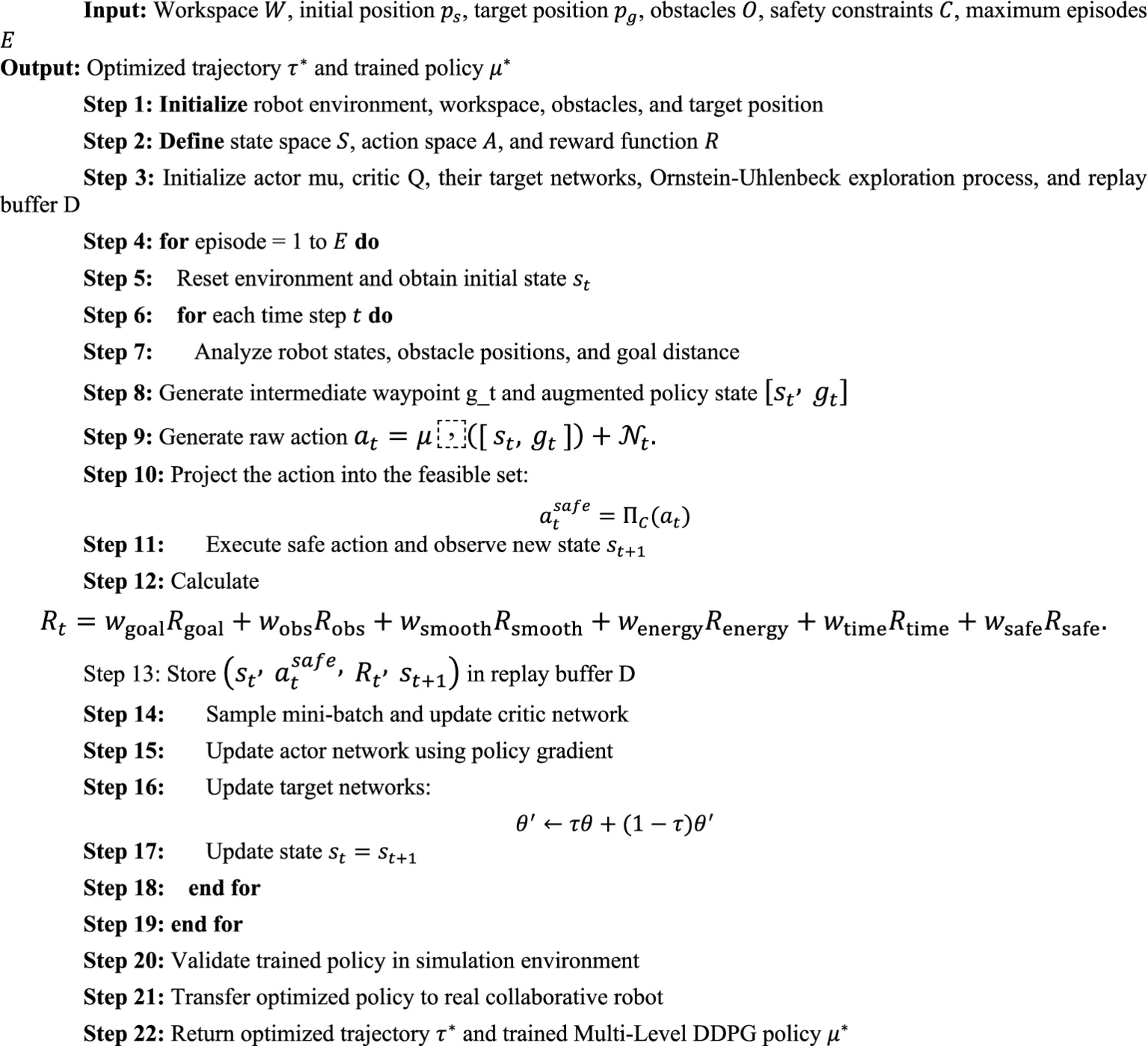

Algorithm 1 therefore trains one off-policy continuous controller while enforcing strategic guidance and reactive safety at every step. This corrected description is consistent with the reward definition in Section 3.4 and Figure 3.

**Figure 3:**
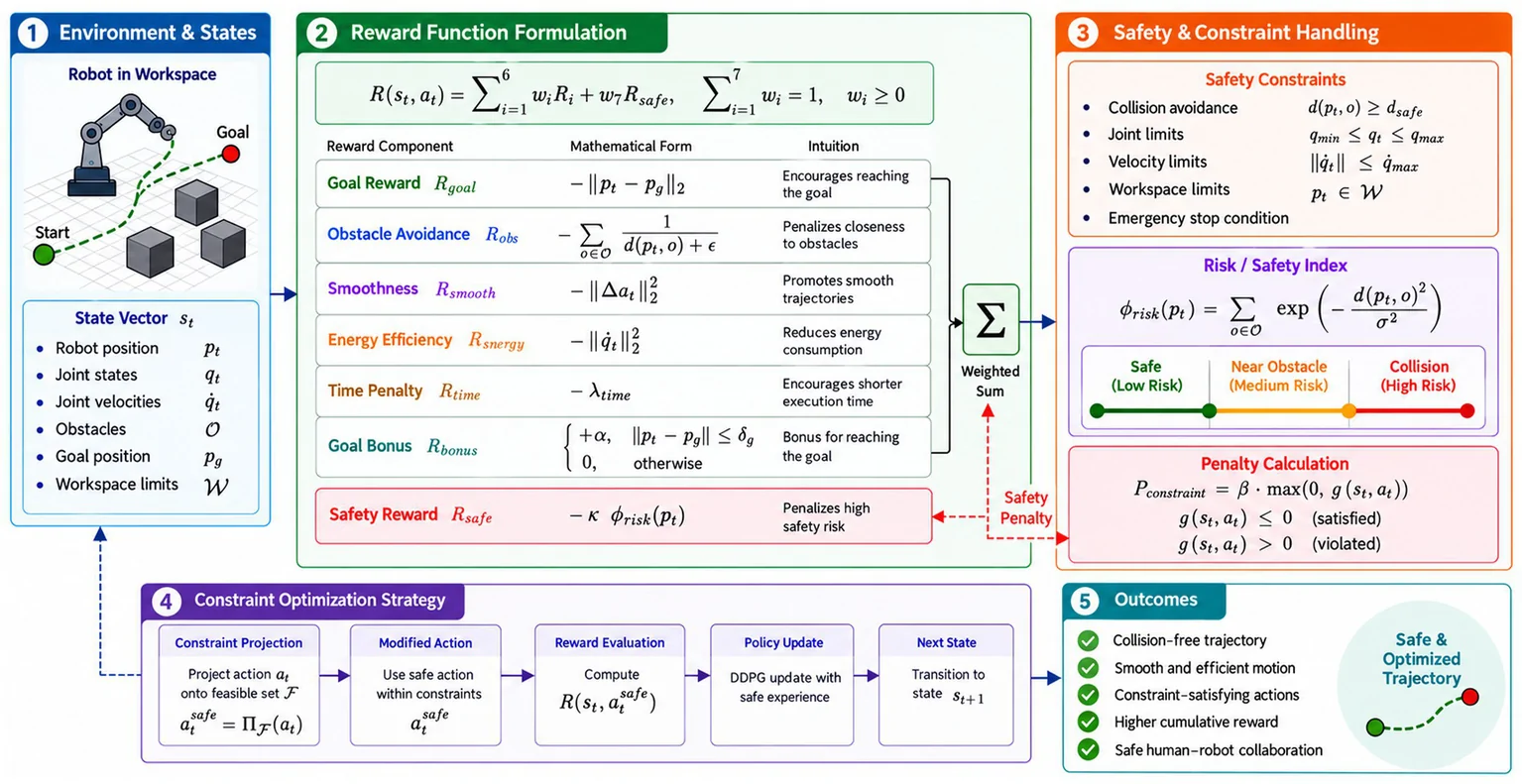
Safety-aware reward function and constraint optimization strategy.

### 3.4 Safety-aware reward function design

The reward comprises the same six components in the text, Figure 3, and Algorithm 1: goal progress, obstacle clearance, action smoothness, mechanical energy, execution time, and safety-constraint satisfaction. All component weights are non-negative, and normalization is required before aggregation so that differently scaled terms do not dominate the update.

The total reward function is mathematically represented as:


$$\begin{array}{l} R(s_{t},a_{t})=w_{1}R_{\text{goal}}+w_{2}R_{\mathrm{obs}}+w_{3}R_{\text{smooth}}\\ +w_{4}R_{\text{energy}}+w_{5}R_{\text{time}}+w_{6}R_{\text{safe}} \end{array}$$

where $w_{i}$ represents the weighting coefficient of each reward component. The goal-reaching reward minimizes the distance between the current robot position and target position:


$$R_{\text{goal}}=-{\left\|p_{t}-p_{g}\right\|}_{2}$$

where $p_{t}$ is the current position and $p_{g}$ represents the desired goal position. The smoothness reward reduces sudden motion changes and improves trajectory stability:


$$R_{\text{smooth}}=-{\left\|a_{t}-a_{t-1}\right\|}^{2}$$

The safety component penalizes unsafe movements and prevents collision conditions:


$$R_{\text{safe}}=-\lambda \sum_{i=1}^{N}\max{\left(0,g_{i}(s_{t},a_{t})\right)}^{2}$$

where $g_{i}(s_{t},a_{t})$ represents the constraint violation function and λ denotes the penalty coefficient.

The overall reward function combines six normalized components corresponding to goal progress, obstacle avoidance, trajectory smoothness, energy efficiency, execution time, and safety constraint satisfaction:


$$\begin{array}{l} R_{t}=w_{\text{goal}}R_{\text{goal}}+w_{\mathrm{obs}}R_{\mathrm{obs}}+w_{\text{smooth}}R_{\text{smooth}}\\ +w_{\text{energy}}R_{\text{energy}}+w_{\text{time}}R_{\text{time}}+w_{\text{safe}}R_{\text{safe}}. \end{array}$$

All reward components are normalized to comparable numerical ranges before aggregation. The weighting coefficients were empirically selected during preliminary training to balance task completion efficiency and safe robot operation. The coefficients used throughout all experiments are


$$\begin{array}{l} w_{\text{goal}}=1.0,w_{\mathrm{obs}}=0.8,w_{\text{smooth}}=0.5,w_{\text{energy}}\\ =0.2,w_{\text{time}}=0.2,w_{\text{safe}}=1.2. \end{array}$$

Safety constraint violations are penalized according to


$$R_{\text{safe}}=-\lambda \;\phi (s_{t},a_{t}),$$

where $\phi (s_{t},a_{t})$ denotes the constraint violation function and the penalty coefficient is fixed to λ=10.

This configuration prioritizes collision-free trajectory generation while simultaneously encouraging accurate goal reaching, smooth motion, energy efficiency, and reduced execution time.

### 3.5 Constraint optimization and policy learning

The reactive level realizes $\Pi_{C}$ as a minimum-deviation constrained projection. For raw action $a_{t}$ , it solves $a_{t}^{\text{safe}}=\arg\;\min u∥u-a_{t}{∥}_{2}^{2}.$ subject to the one-step joint, velocity, workspace, and obstacle-clearance constraints. The projection is evaluated before every environment or hardware command.


$$\begin{array}{cc} a_{t}^{\text{safe}}=\arg\;\min u & ∥u-a_{t}{∥}_{2}^{2}\\ \text{subject to} & q_{\min}\leq q_{t}+\mathrm{\Delta t}\;u\leq q_{\max},\\ & |u|\leq v_{\max},\\ & p(q_{t}+\mathrm{\Delta t}\;u)\in W,\\ & d(p(q_{t}+\mathrm{\Delta t}\;u),O_{t})\geq d_{\text{safe}}. \end{array}$$

Here *p*(.) is forward kinematics, W is the admissible workspace, $O_{t}$ is the current obstacle set, and

$d_{\text{safe}}$ is the configured clearance. The action is first clipped to joint and velocity limits, then the quadratic projection is solved for workspace and clearance constraints. If the problem is infeasible or misses the controller deadline, the command is replaced by a zero-velocity emergency action and the event is counted as a constraint violation.

During training, experiences are stored in replay memory:


$$D=\{(s_{t},a_{t},r_{t},s_{t+1})\}$$

The critic network is optimized by minimizing the temporal difference error:


$$L=\frac{1}{N}\sum_{i}{\left(y_{i}-Q(s_{i},a_{i}|\theta^{Q})\right)}^{2}$$

where the target value is calculated as:


$$y_{i}=r_{i}+\gamma Q^{\prime }(s_{i+1},\mu^{\prime }(s_{i+1})|\theta^{Q^{\prime }})$$

The actor is updated by the deterministic policy gradient, while target networks use the soft update shown above. Critic targets are computed from the projected next action so that value learning reflects commands that can actually be executed. All DDPG-family baselines use the same episode budget, maximum steps, batch size, replay capacity, and evaluation workspace.

### 3.6 Simulation-to-real deployment strategy

After simulation training, the policy was exported to the UR5e navigation platform through the robot-controller interface shown in Figure 4. State and action scaling, joint bounds, workspace coordinates, and controller timing were matched before execution. The present record does not contain a controlled ablation of domain randomization or system identification; therefore, the deployment is reported as an empirical transfer demonstration rather than proof of general sim-to-real robustness. Earlier studies used dynamics or domain randomization for simulation-to-real transfer of robotic control (Peng et al., 2018; Tobin et al., 2017).

**Figure 4:**
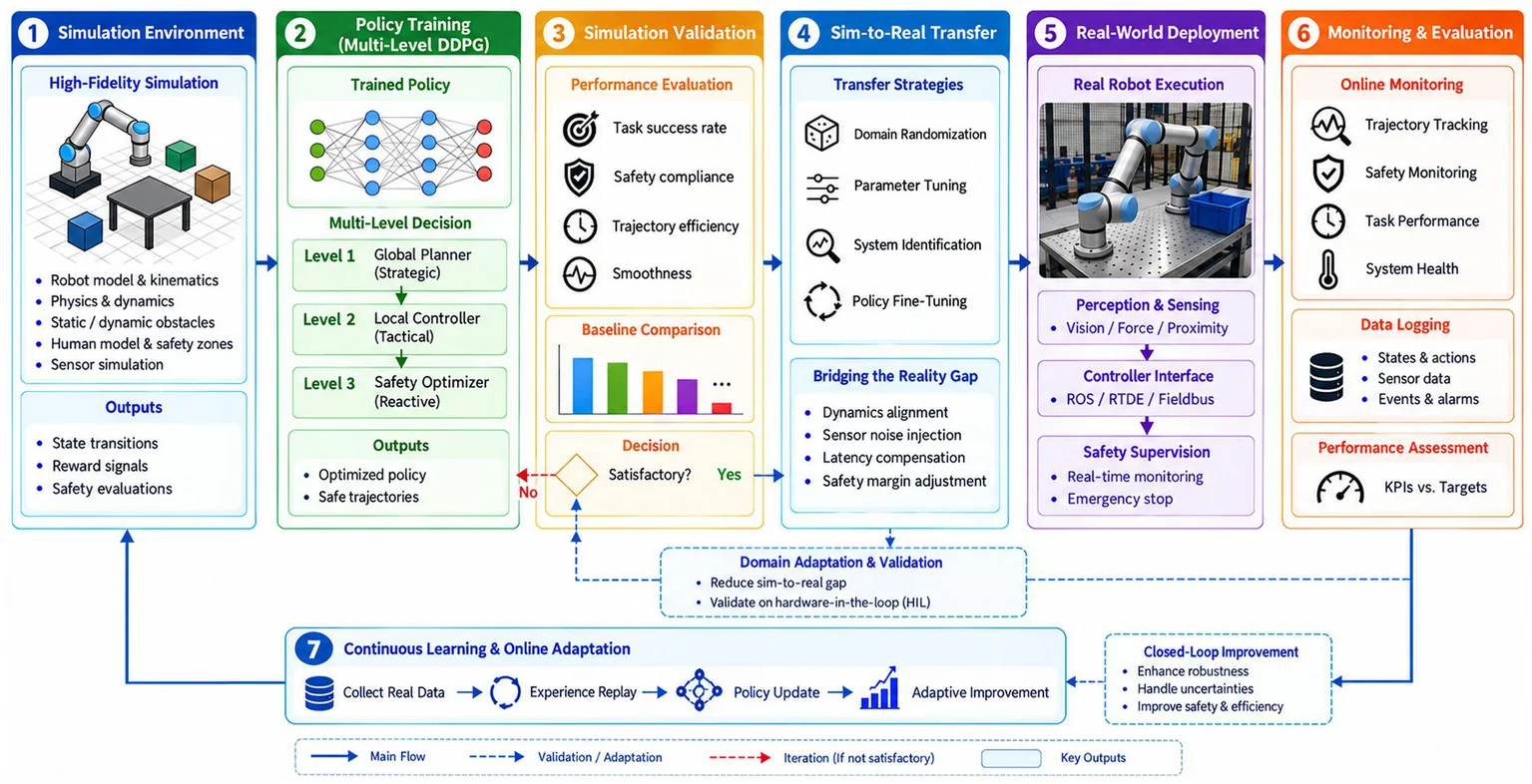
Simulation-to-real deployment framework for collaborative robot control.

The final optimized trajectory is represented as:


$$\tau^{*}=\{p_{1},p_{2},\ldots ,p_{n}\}$$

where each trajectory point satisfies:


$$C(\tau^{*})=\{d_{\mathrm{obs}}>d_{\text{safe}},q_{\min}\leq q_{t}\leq q_{\max},v_{t}\leq v_{\max}\}$$

The quantitative real-world trajectory experiment in Figure 5 uses a UR5e collaborative robot with a gripper. Figure 6 shows a different ABB six-axis manipulator in a welding cell. The ABB sequence is a separate feasibility demonstration and is not evidence that the same policy transferred unchanged between robot morphologies. The exact ABB model identifier was not retained in the available experimental record, which limits hardware reproducibility.

**Figure 5:**
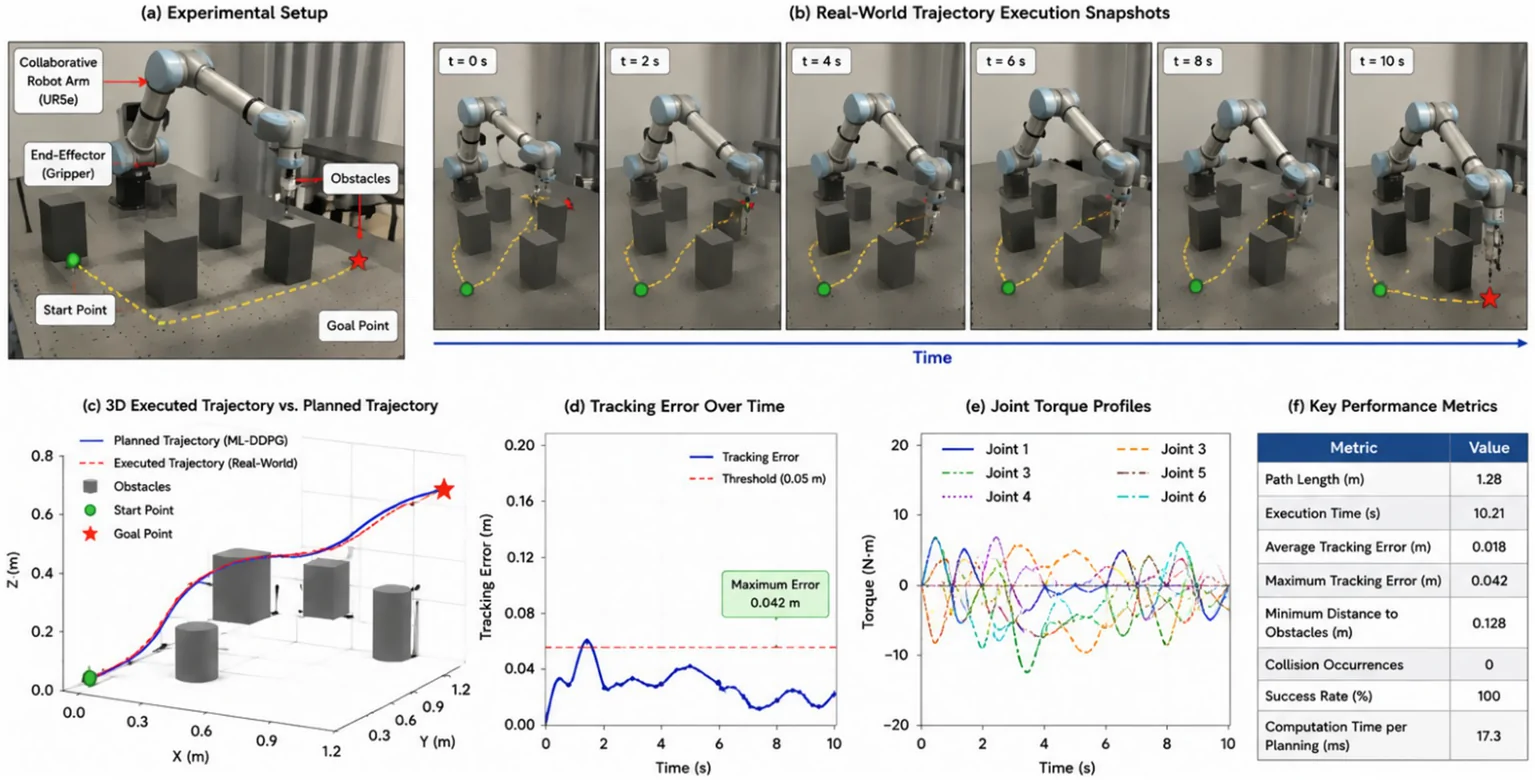
Real-world deployment of ML-DDPG on a UR5e collaborative robot: **(a)** experimental setup; **(b)** trajectory-execution snapshots at 0, 2, 4, 6, 8 and 10 s; **(c)** planned and executed three-dimensional trajectories; **(d)** tracking error over time; **(e)** joint-torque profiles; and **(f)** key performance metrics for the single demonstration.

**Figure 6:**
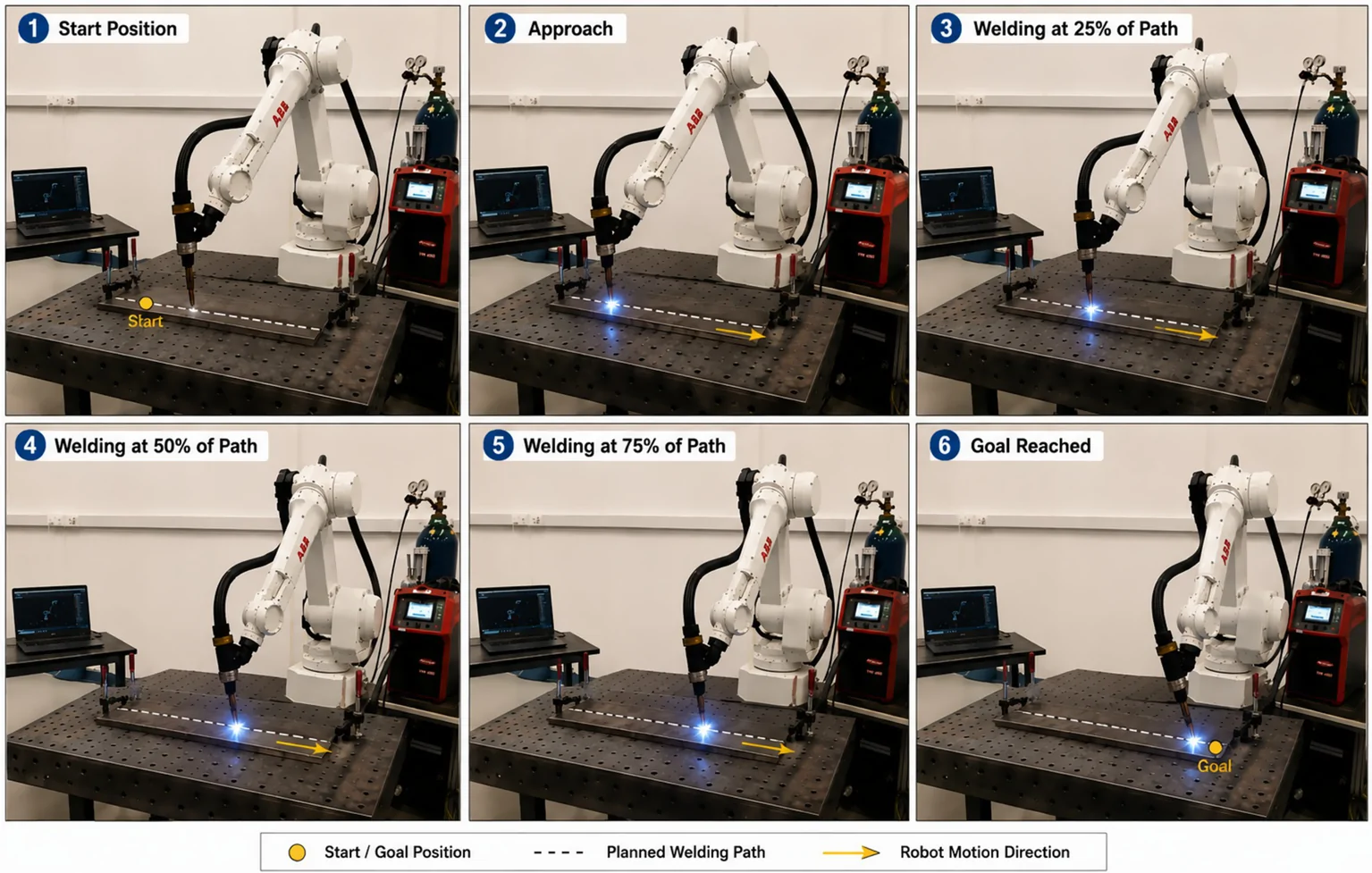
Experimental collaborative robot welding setup for real-world validation of the proposed ML-DDPG framework.

### 3.7 Experimental implementation, metrics, and statistical scope

Table 1 reports the available implementation record: a custom PyTorch-based 3D environment, 200 training episodes, 500 steps per episode, a $1\times 10^{6}$ replay buffer, batch size 64, and the stated learning rates. The navigation and tracking configuration is based on UR5e kinematics. The welding image sequence uses a separate ABB platform and is treated qualitatively.

For a sampled end-effector path ${\left\{p_{i}\right\}}_{i=1}^{N}$ , the evaluation metrics are defined as follows:


$$L=\sum_{i=2}^{N}∥p_{i}-p_{i-1}{∥}_{2},$$

$$E_{\text{traj}}=\frac{1}{N}\sum_{i=1}^{N}∥p_{i}-p_{i}^{\mathrm{ref}}{∥}_{2},$$

$$J=\frac{1}{N-3}\sum_{i=1}^{N-3}∥\Delta^{3}p_{i}{∥}_{2}^{2},$$

$$\mathrm{SI}=\frac{1}{1+J},$$

where identical temporal and spatial normalization is applied to all compared methods.

The mechanical energy consumption is calculated as


$$E_{\text{mech}}=\int_{0}^{T}\sum_{j=1}^{n}|\tau_{j}(t)\;{\dot{q}}_{j}(t)|\mathrm{dt}.$$

The collision rate is defined as


$$R_{\text{collision}}=\frac{N_{\text{collision}}}{N_{\text{episodes}}}\times 100\%,$$

Where $N_{\text{collision}}$ the number of evaluated episodes containing at least one contact.

The success rate is defined as


$$R_{\text{success}}=\frac{N_{\text{success}}}{N_{\text{episodes}}}\times 100\%,$$

Where $N_{\text{success}}$ notes the number of episodes in which the goal is reached without collision or constraint-triggered termination.

The available archive contains one training trace per method and point estimates rather than independent-seed summaries. No standard deviation, confidence interval, or inferential significance test can therefore be reported without new runs. The smoothing rule used to export Figure 7 was also not retained. All comparisons in this manuscript are consequently descriptive; a reproducible resubmission should report at least five independent seeds, the smoothing window, and the number of held-out evaluation episodes per seed.

**Figure 7:**
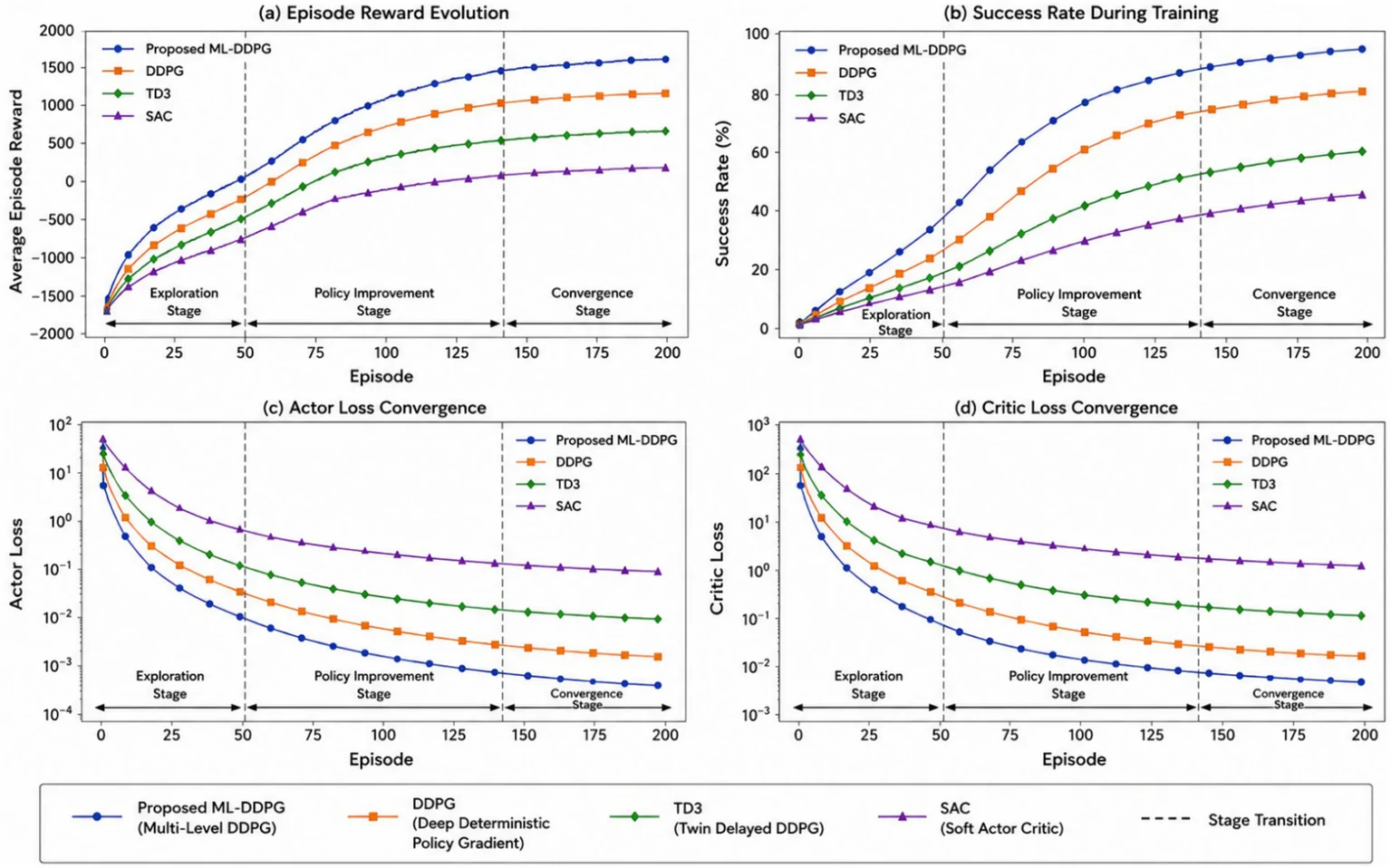
Single-run training traces for ML-DDPG, DDPG, TD3 and SAC over 200 episodes: **(a)** average episode reward; **(b)** success rate; **(c)** actor loss; and **(d)** critic loss.

## 4 Results

The ML-DDPG framework was evaluated for convergence behavior, trajectory quality, safety, dynamic motion, component contribution, and hardware feasibility. Because the retained results are single-run point estimates, the following comparisons describe the supplied experimental record and do not establish statistical significance. Unless otherwise stated, all quantitative results reported in this study correspond to a representative single training run conducted under the experimental protocol described in Section 4. The reported values are presented as descriptive observations of the proposed framework under the specified experimental conditions and should not be interpreted as estimates of statistical variability across repeated independent training runs.

The reward weighting coefficients and safety penalty coefficient listed in Table 1 correspond to the configuration used in the reported experiments and are identical to those defined in Section 3.4. These values were empirically selected during preliminary training and remained fixed throughout all reported evaluations.

### 4.1 Training convergence analysis

Figure 7 presents the exported training traces for ML-DDPG, DDPG, TD3, and SAC. In the retained ML-DDPG run, reward rises more rapidly and becomes visually stable after approximately 140 episodes. This is descriptive stabilization within the shown budget, not a formal convergence proof. The single traces do not support a claim of statistically significant superiority.

The single-run success-rate trace reaches 98.7% at the end of the reported ML-DDPG training run, while the displayed actor and critic losses decrease over training. This value is an observation from one seed, not a mean across independent runs. Because the export record does not identify a rolling-average or other smoothing kernel, Figure 7 should be interpreted as the supplied rendered trace; variability and statistical significance cannot be estimated without new multi-seed experiments.

### 4.2 Trajectory optimization performance

Figure 8 demonstrates the evolution of trajectory generation during different training stages. During the exploration stage shown in Figure 8a, the agent generates highly irregular trajectories with significant deviations and inefficient obstacle avoidance behavior. The generated paths frequently approach obstacles and exhibit excessive motion variations, reflecting the absence of learned navigation knowledge.

**Figure 8:**
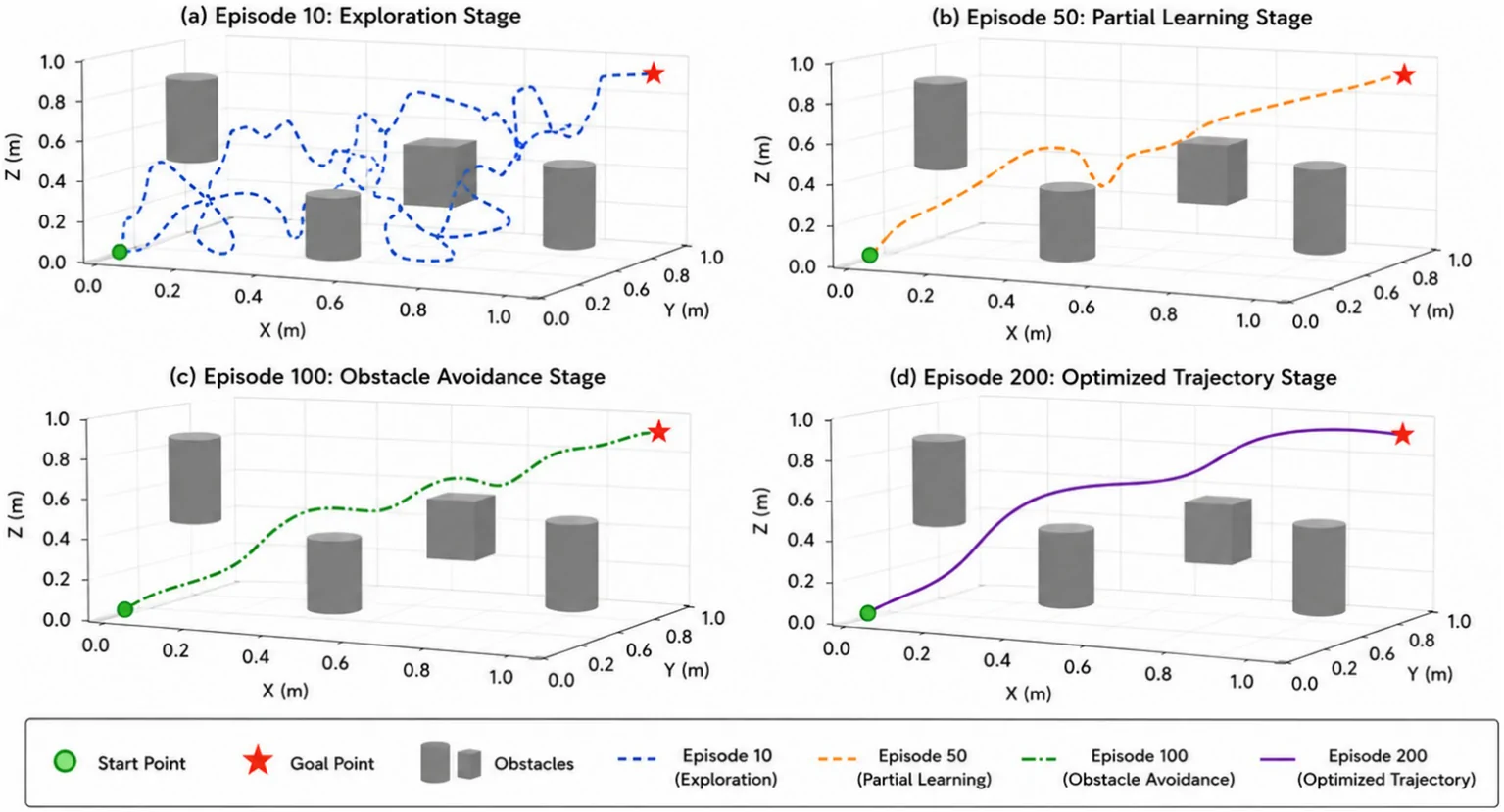
Evolution of trajectory optimization at four training stages: **(a)** episode 10, exploration; **(b)** episode 50, partial learning; **(c)** episode 100, obstacle avoidance; and **(d)** episode 200, optimized trajectory.

Figure 8b represents the partial learning stage, where the agent begins to identify feasible routes toward the target while still exhibiting trajectory deviations and occasional close obstacle interactions. Figure 8c demonstrates substantial performance improvement, with the learned policy producing smoother trajectories and more effective obstacle avoidance. Figure 8d depicts the final optimized trajectory generated after convergence. The trajectory follows a smooth and efficient path, maintains safe distances from all obstacles, and reaches the target without unnecessary deviations. These observations indicate that the proposed ML-DDPG framework progressively learns optimal navigation behavior through continuous interaction with the environment.

### 4.3 Quantitative comparison of trajectory optimization methods

Table 2 reports single-run point estimates for the evaluated planners. In that retained run, ML-DDPG has the shortest reported path length (1.27 m), lowest trajectory error (1.08 mm), highest smoothness index (0.98), no recorded collision, and a 98.7% success rate. These observations are not presented as means over independent seeds and do not support inferential claims.

Figure 9 visualizes the recorded trajectories. Linear interpolation provides limited obstacle adaptation, while A* and RRT* contain sharper directional changes in this workspace. The DDPG-family paths are smoother, and the ML-DDPG trace has the most direct route among the displayed trajectories. The previous external citation attached to this manuscript-generated comparison has been removed.

**Figure 9:**
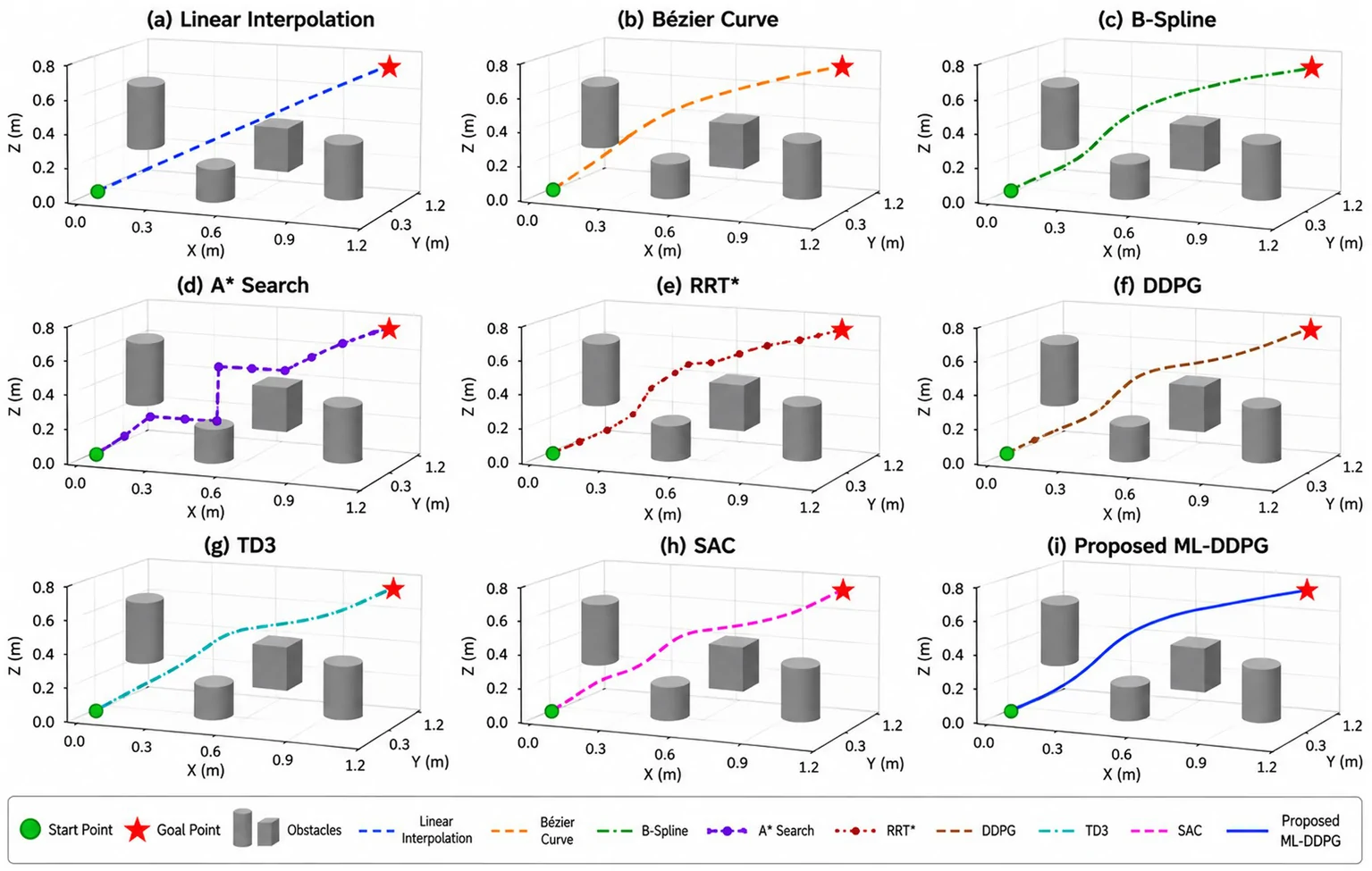
Three-dimensional trajectory comparison: **(a)** linear interpolation; **(b)** Bezier curve; **(c)** B-spline; **(d)** A* search; **(e)** RRT*; **(f)** DDPG; **(g)** TD3; **(h)** SAC; and **(i)** the proposed ML-DDPG.

### 4.4 Safety performance evaluation

Safety is a critical requirement for collaborative robotic systems operating in industrial environments. Figure 10 presents the safety performance analysis of the proposed reward optimization strategy. Figure 10a demonstrates that the collision rate continuously decreases throughout training and approaches zero for the proposed ML-DDPG framework. The reduction in collision events indicates that the safety-aware reward function successfully encourages obstacle avoidance behavior.

**Figure 10:**
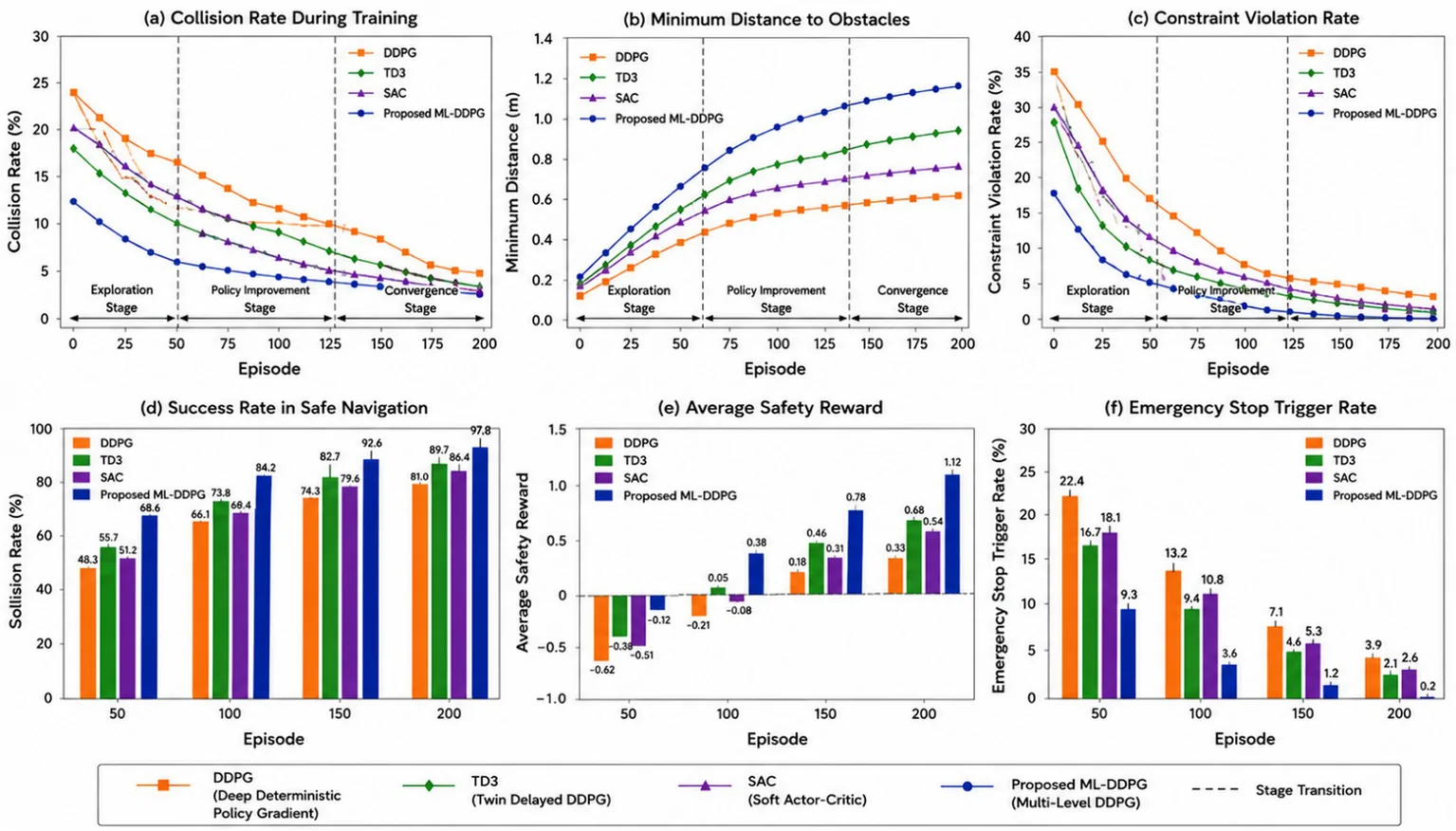
Safety performance over 200 episodes: **(a)** collision rate; **(b)** minimum distance to obstacles; **(c)** constraint-violation rate; **(d)** safe-navigation success rate; **(e)** average safety reward; and **(f)** emergency-stop trigger rate for ML-DDPG and the baselines.

Figure 10b shows the minimum distance maintained between the robot and surrounding obstacles. The proposed framework consistently achieves larger obstacle clearance compared with other reinforcement learning approaches, indicating more conservative and safer navigation decisions. Figure 10c further illustrates a substantial reduction in constraint violation rates as training progresses. The proposed approach achieves the lowest violation frequency, confirming the effectiveness of the constraint optimization mechanism.

The bar-chart analysis presented in Figures 10d–f further supports these observations. The proposed ML-DDPG achieves the highest safe navigation success rate, the largest average safety reward, and the lowest emergency stop activation frequency. Collectively, these results demonstrate that the proposed reward optimization strategy effectively balances task completion and safety requirements during trajectory generation.

### 4.5 Dynamic motion profile analysis

Figure 11 compares the dynamic motion characteristics generated by different planning approaches. Figure 11a demonstrates the linear velocity profiles during trajectory execution. The proposed ML-DDPG maintains a stable velocity profile with smooth acceleration and deceleration phases, reducing sudden motion changes. Figures 11b,c illustrate angular velocity and acceleration behaviors, respectively. The proposed framework produces significantly fewer oscillations and lower acceleration peaks than competing approaches.

**Figure 11:**
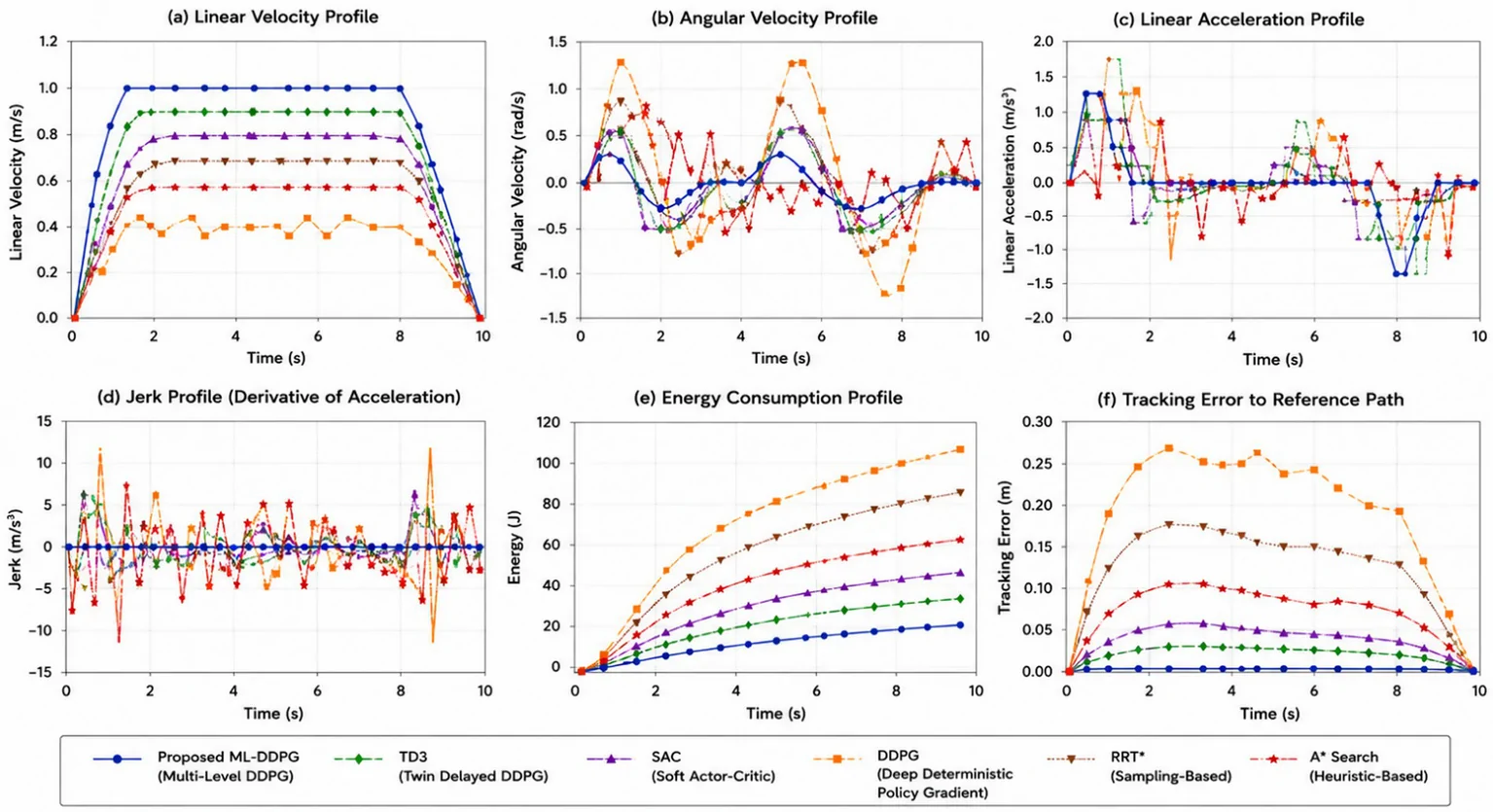
Dynamic motion profiles over 10 s: **(a)** linear velocity; **(b)** angular velocity; **(c)** linear acceleration; **(d)** jerk; **(e)** energy consumption; and **(f)** trajectory tracking error for ML-DDPG and the comparison methods.

Figure 11d presents the jerk profiles, which represent the derivative of acceleration and provide an important indicator of motion smoothness. The proposed ML-DDPG exhibits the smallest jerk variations, indicating improved dynamic feasibility and reduced mechanical stress on robotic components. Figure 11e shows cumulative energy consumption during task execution. The optimized trajectory generated by the proposed framework requires the lowest energy expenditure, demonstrating improved efficiency. Figure 11f further reveals the lowest trajectory tracking error among all evaluated methods. These results confirm that the proposed framework generates dynamically feasible trajectories suitable for practical industrial deployment.

### 4.6 Ablation analysis

Table 3 presents the ablation study conducted to evaluate the contribution of individual components within the proposed framework. The baseline DDPG model achieves a success rate of 93.1% and a trajectory error of 3.42 mm. The introduction of the multi-level control strategy improves success rate and reduces trajectory error, indicating that hierarchical decision-making contributes to more effective navigation behavior.

**Table 3:** Single-run ablation analysis of the proposed ML-DDPG framework.

Model variant	Success rate (%)	Trajectory error (mm)	Collision rate (%)	Smoothness index ↑	Average reward	Planning time (ms)
Baseline DDPG	93.1	3.42	3.6	0.91	1124.7	18.7
+ Multi-level control	95.2	2.67	2.4	0.94	1286.3	18.1
+ Safety-aware reward	96.8	2.03	1.2	0.95	1398.5	17.8
+ Constraint optimization	97.9	1.42	0.4	0.97	1517.8	17.4
Full ML-DDPG framework	98.7	1.08	0.0	0.98	1638.4	17.1

Within the retained single-run ablation record, adding the safety-aware reward is associated with fewer observed collisions and a higher reward, while adding the constraint-projection module is associated with fewer safety violations and a higher smoothness index. The complete ML-DDPG configuration produced observed values of 98.7% success, 1.08 mm trajectory error, a 0% collision rate, and the highest reward in that run. These measurements are descriptive single-run observations; they do not establish the statistical contribution of each component without independent-seed repetition.

### 4.7 Real-world deployment evaluation

Figure 5 demonstrates the deployment of the proposed ML-DDPG framework on a collaborative robot operating in a real industrial environment. Figure 5a presents the experimental setup, including obstacle placement, start location, and target destination. Figure 5b depicts sequential trajectory execution snapshots, illustrating successful navigation around obstacles while maintaining smooth motion behavior.

Figure 5 reports the UR5e navigation and tracking demonstration. The planned and executed trajectories remain close in the displayed trial; the embedded summary reports a 1.28 m path, 10.21 s execution time, 0.018 m average tracking error, 0.042 m maximum tracking error, 0.128 m minimum obstacle distance, no collision, and 17.3 ms computation time per planning step. These values quantify the shown demonstration but do not replace repeated hardware trials.

Figure 6 shows a separate ABB welding-cell sequence. In this demonstration, ML-DDPG supplies the Cartesian path reference and waypoint progression, while the robot’s low-level controller executes joint tracking and the welding equipment controls the process. The images document completion of the illustrated path, but synchronized position, velocity, force, current, weld-quality, and repeatability logs were not supplied. The figure is therefore qualitative feasibility evidence only; it does not establish quantitative welding accuracy, safety certification, or policy transfer across the UR5e and ABB platforms.

## 5 Discussion

The descriptive results suggest that coupling strategic waypoints, one continuous DDPG policy, and a reactive safety projection can reduce decision burden relative to an unstructured single-level controller. This interpretation differs from option-based hierarchical reinforcement learning, which learns temporally extended subpolicies (Sutton et al., 1999), and from fixed phase-based manipulation, which switches among task stages (Navazani et al., 2026). The present architecture uses continuous waypoint conditioning and a shared policy, so its contribution lies in the coupling mechanism rather than hierarchy alone.

The trajectory results also indicate a useful trade-off between path directness, tracking accuracy, smoothness, and planning latency in the evaluated workspace. Classical planners remain important because they offer interpretable geometric behavior, whereas the learned policy adapts its continuous commands from interaction. The reported advantage should nevertheless be understood as environment-specific until identical evaluation protocols are repeated across seeds, obstacle distributions, payloads, and robot platforms.

Safety improvements in the supplied run are consistent with combining reward penalties and explicit action projection. The projection can prevent a raw command from violating modeled bounds even when the policy is imperfect, which complements objective-level constraints (Achiam et al., 2017; Dalal et al., 2018). However, no recorded collision in one evaluation is not equivalent to a probabilistic safety guarantee or certification for human-robot collaboration.

Several limitations constrain generalization. The retained archive includes one training seed per method and does not preserve the smoothing rule or evaluation-trial count. The numerical reward-weight vector and safety penalty coefficient used in the experiments are fully reported in Section 3.4 and Table 1. The quantitative hardware evidence is limited to the UR5e demonstration, whereas the ABB welding sequence is qualitative and uses a different robot morphology. The study does not test additional collaborative robots, multiple human motion patterns, sensor occlusion, payload variation, network delay, or multi-robot operation. These limitations preclude broad statistical or cross-platform claims.

Potential failure cases include projection infeasibility in narrow passages, repeated action clipping near joint limits, delayed obstacle observations, out-of-distribution obstacle motion, calibration drift, controller latency, and reward trade-offs that favor task progress over clearance. Future work should report five or more independent seeds, confidence intervals and effect sizes, repeated hardware trials on multiple cobot models, ablation of each sim-to-real mechanism, dynamic human-in-the-loop scenarios, and formally verified fallback control.

## 6 Conclusion

This study presents ML-DDPG as a coupled three-level trajectory-planning framework comprising strategic waypoint generation, one learned DDPG controller, and reactive constrained action projection. In the retained single training run, the framework produced observed values of 98.7% success, 1.08 mm trajectory error, a 0.98 smoothness index, and no recorded collision. These values are single-run observations without estimates of variability and therefore do not establish statistical superiority. The UR5e experiment provides quantitative evidence from one demonstration, while the separate ABB welding sequence provides qualitative feasibility evidence only. The results support further investigation but do not establish certified safety or robot-independent transfer. The principal limitations are the single-seed training record, unavailable variability estimates, an unpreserved smoothing rule and evaluation-trial count, limited quantitative hardware trials, and the absence of diverse cobot and human-interaction scenarios. Future research will prioritize multi-seed statistical evaluation, complete reporting of evaluation procedures, repeated tests on additional collaborative robot platforms, dynamic human-aware constraints, and verified emergency fallback mechanisms.

## Data Availability

The original contributions presented in the study are included in the article/supplementary material, further inquiries can be directed to the corresponding author.
